# Impact of changes in mode of travel to work on changes in body mass index: evidence from the British Household Panel Survey

**DOI:** 10.1136/jech-2014-205211

**Published:** 2015-05-07

**Authors:** Adam Martin, Jenna Panter, Marc Suhrcke, David Ogilvie

**Affiliations:** 1 Health Economics Group and UKCRC Centre for Diet and Activity Research (CEDAR), Norwich Medical School, University of East Anglia, Norwich, UK; 2 MRC Epidemiology Unit and UKCRC Centre for Diet and Activity Research (CEDAR), University of Cambridge, Cambridge, UK; 3 Centre for Health Economics, University of York, York, UK

**Keywords:** PHYSICAL ACTIVITY, WORKPLACE, OBESITY

## Abstract

**Background:**

Active commuting is associated with various health benefits, but little is known about its causal relationship with body mass index (BMI).

**Methods:**

We used cohort data from three consecutive annual waves of the British Household Panel Survey, a longitudinal study of nationally representative households, in 2004/2005 (n=15 791), 2005/2006 and 2006/2007. Participants selected for the analyses (n=4056) reported their usual main mode of travel to work at each time point. Self-reported height and weight were used to derive BMI at baseline and after 2 years. Multivariable linear regression analyses were used to assess associations between switching to and from active modes of travel (over 1 and 2 years) and change in BMI (over 2 years) and to assess dose–response relationships.

**Results:**

After adjustment for socioeconomic and health-related covariates, the first analysis (n=3269) showed that switching from private motor transport to active travel or public transport (n=179) was associated with a significant reduction in BMI compared with continued private motor vehicle use (n=3090; −0.32 kg/m^2^, 95% CI −0.60 to −0.05). Larger adjusted effect sizes were associated with switching to active travel (n=109; −0.45 kg/m^2^, −0.78 to −0.11), particularly among those who switched within the first year and those with the longest journeys. The second analysis (n=787) showed that switching from active travel or public transport to private motor transport was associated with a significant increase in BMI (0.34 kg/m^2^, 0.05 to 0.64).

**Conclusions:**

Interventions to enable commuters to switch from private motor transport to more active modes of travel could contribute to reducing population mean BMI.

## Introduction

Public health guidelines encourage adults to undertake at least 30 min of moderate-intensity physical activity daily to help prevent obesity and several other chronic conditions.^1^ While opportunities to increase time spent being active at home or during leisure or work time can be costly or limited, incorporating walking or cycling into the journey to and from work may represent a relatively low cost, more feasible option for many people.^2–4^ Cross-sectional studies have identified individual-level associations between walking and cycling to work and various health outcomes including lower body mass index (BMI)^5^ ^6^ and lower prevalence of cardiovascular disease or diabetes.^5^ ^7^ Of 30 individual-level studies of the association between active travel and BMI identified in a recent review, 25 reported statistically significant negative relationships (p<0.05).^8^ However, just one study identified in the review,^9^ and one further study of the relationship between active travel and overall physical activity in adults,^10^ used longitudinal study designs. This limits the potential for drawing reliable causal inferences, not least because other studies have indicated that increases in body weight may precede reductions in physical activity.^8^ ^11^ ^12^ Other longitudinal ecological studies have demonstrated population-level correlations between decreasing active travel,^13^ increasing car use^14–16^ and increasing prevalence of adult obesity or average BMI over time. To the best of our knowledge, however, no longitudinal study has used a nationally representative data set to examine the individual-level impact on BMI of switching between modes of travel.^17^ This paper uses cohort data from the British Household Panel Survey (BHPS) to estimate the effects on BMI of switching between private motor transport and active travel or public transport (which typically involves some walking or cycling to or from stations or stops)^18^ ^19^ for the journey to and from work.

## Methods

### British Household Panel Survey

The BHPS is a longitudinal study of private households in Great Britain that began in 1991/1992 as an annual survey of each adult member of a nationally representative sample of households (BHPS waves after 2008/2009 are encompassed in the new ‘Understanding Society’ survey, http://www.iser.essex.ac.uk/survey/bhps).^20^ Self-reported height and weight were reported in only two waves: September 2004–May 2005 (subsequently referred to as t0, n=15 791) and September 2006–March 2007 (t2, n=15 392). Data from these two waves and an intermediate wave (t1, September 2005–May 2006) were used in these analyses. Participants consented to use their survey information, and the data for these analyses were anonymous, with access administered by the UK Data Archive (http://www.data-archive.ac.uk).

### Sample selection

**Figure 1:**
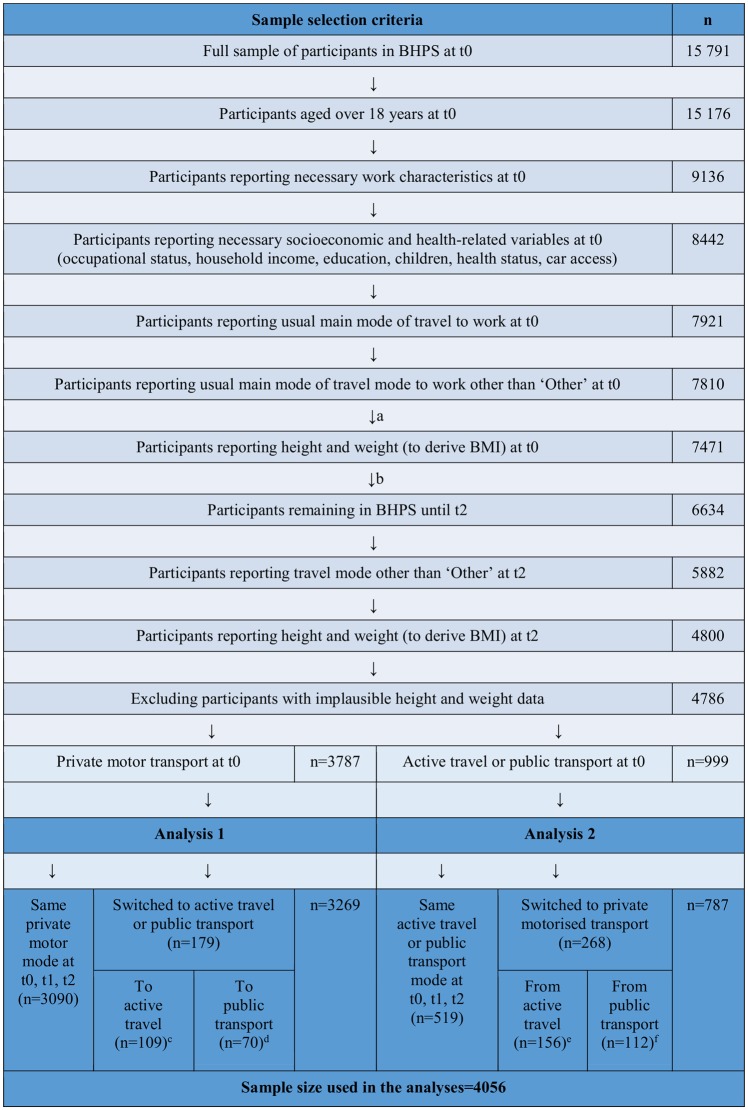
Samples used in the analyses and description of sample selection criteria. (a) To assess missing variables bias (height and weight data), characteristics of individuals who reported travel mode other than ‘other’ at t0 but not height and weight at t0 are compared with individuals who remained in the sample (see online supplementary appendix). (b) To assess attrition bias, characteristics of individuals who reported height and weight at t0 but had dropped out of the sample before t2 are compared with individuals who remained in the sample (see online supplementary appendix). (c) Of whom 10 participants had a commute time of >30 min at t0, 42 switched between t0 and t1, and the most common travel mode switched to was walking (n=83). (d) Of whom 32 participants had a commute time of >30 min at t0, 26 switched between t0 and t1, and the most common travel mode switched to was rail travel (n=32). (e) Of whom 10 participants had a commute time of >30 min at t0, 84 switched between t0 and t1, and the most common travel mode switched from was walking (n=121). (f) Of whom 59 participants had a commute time of >30 min at t0, 56 switched between t0 and t1, and the most common travel mode switched from was bus/coach travel (n=73).

Figure 1 shows how the sample used in the analyses (n=4056) was selected from the original BHPS sample at t0 (n=15 791). Participants eligible for inclusion in the analyses were those aged over 18 years who reported the socioeconomic and health status characteristics listed below (under ‘Covariates and other participant characteristics’) and who reported their usual main mode of travel to work, height and weight at t0 and t2. An assessment of attrition bias and missing values bias comparing participants in the original BHPS sample with those retained in the analytical sample is presented in the online supplementary appendix.

### Change in BMI

The outcome variable used in the analyses was change in BMI between t0 and t2. BMI in each wave was calculated by dividing self-reported weight (reported in kg, or converted to kg from stones and pounds) by the square of self-reported height (reported in metres, or converted to metres from feet and inches). Where height differed between waves, baseline height was used to prevent small artefactual differences in height affecting the results (if, eg, height was reported using metric units in one wave and imperial units in the other). Follow-up height measures were used to replace implausible baseline values attributable to obvious data entry errors in three cases. A small number of participants were excluded from the analyses due to implausible values for weight (<30 kg, n=7) or change in weight (>87 kg, n=7). Following contact with BHPS administrators, other adjustments were also made for coding errors in imperial measurements (please contact authors for details).

### Change in usual mode of travel to work

Participants reported their usual main mode of travel to work at t0, t1 and t2 in nine categories. For each wave, participants were categorised as using active modes of travel (‘walking’ or ‘cycling’), public transport (‘bus/coach’, or rail: ‘train’ or ‘underground/metro’), or private motor transport (‘car or van’, ‘car/van passenger’ or ‘motorcycle’). Participants who reported using ‘other’ modes of travel were excluded from analysis.

### Covariates and other participant characteristics

Covariates were used to account for selected individual-level characteristics reported at t0, and changes in individual-level characteristics between t0 and t2, which have previously been shown to be associated with active travel and obesity,^5^ ^6^ ^16^ ^21–25^ and hence were hypothesised to act as potential confounders of the relationship between active travel and BMI. The covariates reported at t0 were: age, gender, occupational status (for analytical purposes, binary variables were created for each of the seven Registrar General’s Social Class categories), working hours (2 binary variables: weekly hours of work ≥30 (‘full-time’) versus <30 (‘part-time’), and night-time versus other-time work), annual household income (quintiles to account for the impact of household size and age of children on living standards, using the McClements equivalence scale),^20^ educational level (degree or higher qualification vs less than degree), number of children under 16 in the household (one or more vs none), self-reported health status (5 categories from ‘excellent’ to ‘very poor’), and number of cars in the household (1 or more vs none). The covariates which accounted for changes that occurred between t0 and t2 were: home location (a single variable: ≥1 move between t0 and t2), household income (2 variables: increase and decrease of >2 quintiles), health status (2 variables: increase and decrease of ≥2 categories), car access (2 variables: gaining and losing household access to ≥1 car), pregnancy (2 variables: becoming and no longer being pregnant).

Other variables reported at t0 were also selected for use in the descriptive statistics: commuting time (minutes), region (13 categories), annual frequency of primary care and hospital outpatient visits, smoking status, and frequency of leisure activities in three separate categories: playing sport, walking or swimming (hereafter leisure time physical activity or LTPA), gardening and eating out.

### Statistical analysis

**Figure 2:**
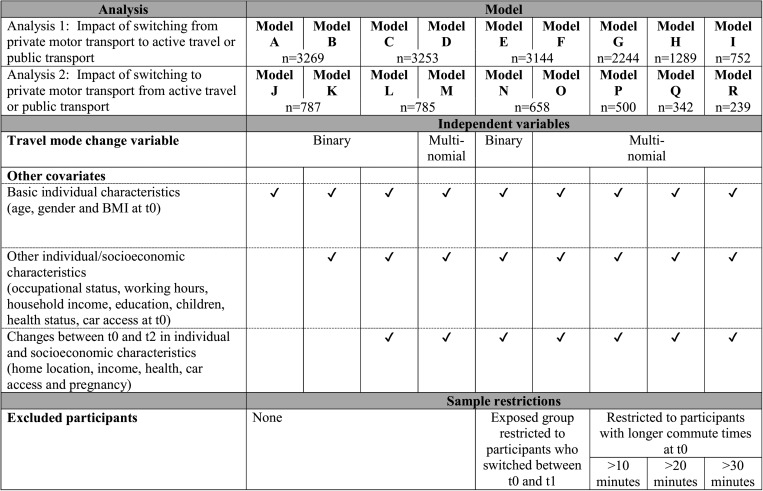
Summary of the independent variables and sample restrictions used in the statistical models.

The variables and subsamples selected for use in 18 separate analytical models (models A-R) are summarised in figure 2. To assess the effects of switching to and from active commuting, two separate analyses were conducted. First, we examined the effect of switching from private motor transport at t0 to active travel or public transport at t2 on change in BMI (analysis 1). Participants who switched (‘the exposed’) were compared with those who maintained use of the same mode of private motor transport at t0, t1 and t2 (‘the unexposed’). Those participants in the exposed group who had switched between t0 and t1 were also compared with those in the unexposed group in order to study temporal effects. Second, we examined the effect of switching from active travel or public transport at t0 to private motor transport at t1 or t2 on BMI (analysis 2). Participants who switched were compared with those who maintained use of the same mode of active travel or public transport at t0, t1 and t2. Participants who switched between different modes of private motor transport (analysis 1) or of active travel or public transport (analysis 2) were excluded from the respective unexposed groups. χ^2^, Mann-Whitney and Student t tests were used to compare the characteristics of the exposed and unexposed groups.

Multivariable linear regression models were used to estimate the association between change in usual mode of transport (binary or multinomial independent variable) and change in BMI with progressive adjustment for (1) individual characteristics (age, gender and BMI at t0), (2) further characteristics at t0 (occupational status, working hours, household income, education, children, health status and car access) and (3) changes in home location, income, health, car access and pregnancy status. Additional analyses were used to explore dose–response relationships using subsamples of participants with different baseline commute times (in 3 separate categories >10, >20 and >30 min), a reasonable proxy for distance to work, since all participants in a given analysis used the same usual mode of travel at t0. Statistical analyses were conducted using STATA V.13.1.

## Results

### Characteristics of the sample

Table 1 shows basic descriptive statistics and comparisons of groups used in the analyses at t0 and t2.

**Table 1:** Descriptive statistics

Descriptive statistics and group comparisons for participants used in analysis 1					
	Unexposed	Switched to active travel		Switched to public transport	
N (minimally adjusted models A and B)†	3090	109		70	
Characteristic (at t0 unless shown otherwise)	Per cent or mean	Per cent or mean	p Value‡	Per cent or mean	p Value‡
*Sociodemographic characteristics*					
Age (mean years)	41.2	37.8**	**0.002**	36.8**	**0.001**
Male§	61.7%	58.7%	0.527	57.1%	0.437
Professional or managerial occupation§	44.1%	41.3%	0.559	41.4%	0.655
Full time work§	85.5%	77.1%*	**0.014**	78.6%	0.103
Works at night time§	2.2%	2.8%	0.701	1.4%	0.662
Household income (mean £)	32 495	28 087 **	**0.002**	35 141	0.460
High income§	45.2%	33.9%*	**0.020**	47.1%	0.748
Education: degree or higher qualification§	19.4%	13.8%	0.139	34.3%**	**0.002**
One or more children in the household§	17.1%	22.0%	0.184	10.0%	0.117
Lives in London or South-East England§	11.7%	10.1%	0.617	18.6%	0.076
*Health related characteristics*					
BMI (mean kg/m^2^)	26.9	26.1	0.056	26.0	0.140
WHO-classified overweight§	64.7%	52.3%**	**0.008**	54.3%	0.071
‘Poor’ or ‘very poor’ self-assessed health§	3.6%	4.6%	0.585	7.1%	0.118
Self-reported smoker§	22.8%	31.2%*	**0.041**	21.4%	0.784
More than 3 annual hospital visits§	10.4%	9.2%	0.675	11.4%	0.785
More than 6 annual primary care visits§	9.1%	8.3%	0.765	10.0%	0.794
*Travel related*					
One or more cars in household§	98.8%	95.4%**	**0.003**	90.0%***	**<0.001**
One or more cars in household (t2)§	99.0%	93.6%***	**<0.001**	80.0%***	**<0.001**
Number of cars in household (mean)	1.8	1.8	0.707	1.4***	**<0.001**
Number of cars in household (t2, mean)†	1.8	1.6**	**0.002**	1.2***	**<0.001**
Private motor transport user in t0–t1 and t0–t2§†	91.9%	70.5%***	**<0.001**	64.4%***	**<0.001**
Commute time (mean minutes)†	23.0	16.5***	**0.001**	33.7***	**<0.001**
Commute time (t2, mean minutes)†	23.6	13.9***	**0.001**	45.8***	**<0.001**
*Other lifestyle-related characteristics*					
At least weekly LTPA§	57.8%	68.8%*	**0.022**	58.6%	0.901
At least weekly LTPA (t2)§	59.2%	78.9%***	**0.001**	68.6%	0.113
At least weekly gardening§	25.8%	17.4%	0.050	14.3%*	**0.029**
At least weekly gardening (t2)§	28.8%	22.0%	0.122	15.7%*	**0.016**
At least weekly eating out§	16.8%	14.7%	0.555	20.0%	0.484
At least weekly eating out (t2)§	16.7%	14.7%	0.578	17.1%	0.922

Descriptive statistics and group comparisons for participants used in analysis 2					
	Unexposed	Switched from active travel		Switched from public transport	
N (minimally adjusted models J and K)†	519	156		112	
Characteristic (at t0 unless shown otherwise)	Per cent or mean	Per cent or mean	p Value‡	Per cent or mean	p Value‡
*Sociodemographic characteristics*					
Age (mean years)	41.2	35.1***	**<0.001**	33.9***	**<0.001**
Male§	49.9%	54.5%	0.315	52.7%	0.594
Professional or managerial occupation§	34.5%	24.4%*	**0.018**	38.4%	0.433
Full time work§	73.0%	71.8%	0.762	77.7%	0.309
Works at night time§	1.7%	0.6%	0.322	0.9%	0.518
Household income (mean £s)	31 829	29 842	0.131	33 865	0.421
High income§	37.2%	32.1%	0.241	39.3%	0.677
Education: degree or higher qualification§	20.4%	13.5%	0.051	19.6%	0.852
One or more children in the household§	17.0%	16.0%	0.785	14.3%	0.490
Lives in London or South-East England§	20.2%	14.1%	0.086	22.3%	0.620
*Health-related characteristics*					
BMI (mean kg/m^2^)	26.1	26.3	0.634	25.7	0.339
WHO-classified overweight§	54.7%	49.4%	0.239	49.1%	0.280
‘Poor’ or ‘very poor’ self-assessed health§	4.2%	4.5%	0.893	7.1%	0.190
Self-reported smoker§	26.6%	27.6%	0.810	27.7%	0.813
More than 3 annual hospital visits§	11.2%	7.7%	0.211	15.2%	0.235
More than 6 annual primary care visits§	10.4%	10.3%	0.958	6.3%	0.177
*Travel related*					
One or more cars in household§	73.4%	81.4%*	**0.042**	72.3%	0.813
One or more cars in household (t2)†§	74.9%	92.3%***	**<0.001**	91.0%***	**<0.001**
Number of cars in household (mean)	1.0	1.2**	**0.001**	1.0	0.624
Number of cars in household (t2, mean)†	1.0	1.5***	**<0.001**	1.4***	**<0.001**
Private motor transport user in t0–1 and t0–2†§	4.6%	22.0%***	**<0.001**	17.8%***	**<0.001**
Commute time (mean minutes)†	27.4	13.7***	**<0.001**	42.4***	**<0.001**
Commute time (t2, mean minutes)†	28.2	18.0**	**0.002**	29.5	0.115
*Other lifestyle-related characteristics*					
At least weekly LTPA§	64.7%	74.4%*	**0.025**	56.3%	0.091
At least weekly LTPA (t2)§	65.5%	65.4%	0.977	65.2%	0.947
At least weekly gardening§	20.2%	21.8%	0.672	17.0%	0.430
At least weekly gardening (t2) §	20.8%	21.2%	0.926	17.9%	0.481
At least weekly eating out§	17.1%	21.2%	0.254	17.9%	0.857
At least weekly eating out (t2)§	15.6%	17.9%	0.486	20.5%	0.202

*p<0.05, **p<0.01 and ***p<0.001.

†Values for some variables were not reported for all individuals included in the minimally adjusted models.

‡The results of χ^2^ test (or Mann-Whitney U test for number of cars, age, income and commute time, or student t test for BMI), where the null hypothesis was that the difference between the exposed and unexposed group was equal to 0.

§Binary variables were created as described in the Methods section. Additionally, binary variables were created for the highest occupational status (professional/managerial=1) compared with all other occupations (=0), the two highest income quintiles (=1) compared with all other income quintiles (=0), resident in London or South East England (=1) compared with all other regions (=0), being classed as overweight or obese (=1) compared with any other weight status (=0), poor or very poor self-assessed health (=1) compared with fair or good self-assessed health (=0), and for three indicators of leisure activities (=1 if undertaken at least once a week,=0 if undertaken less frequently).

BMI, body mass index; LTPA, leisure time physical activity. WHO, World Health Organization.

### Analysis 1: Switching from private motor transport to active travel or public transport

Of 3269 individuals included in this analysis, 179 were in the exposed group. Of these, 109 switched to active travel (most often walking, n=83) and 70 to public transport (most often rail, n=32). Switchers were significantly younger on average than non-switchers (eg, for active travel: 37.8 vs 41.2 years at t0, table 1) and less likely to have access to a car (eg, for active travel: 95.4% vs 98.8%). No statistically significant differences were observed between groups in terms of mean BMI, although those who switched to active travel were less likely to be classified as overweight or obese at baseline (52.3% vs 64.7%). Those who switched to active travel, but not those who switched to public transport, also had a significantly lower mean adjusted household income (£28 087 vs £32 495); a higher likelihood of smoking (31.2% vs 22.8%); a shorter mean commute time (16.5 vs 23.0 min at t0), which became shorter still after taking up active travel (13.9 min at t2); and a higher likelihood of weekly LTPA (68.8% vs 57.8% at t0) than non-switchers. Those who switched to public transport were significantly more likely to hold a degree or higher qualification (34.3% vs 19.4%). No statistically significant differences in household composition or health status were observed between groups.

#### Effect on BMI

Switching from private motor transport to active travel or public transport was associated with a significant reduction in BMI of −0.32 kg/m^2^ (95% CI −0.60 to −0.05) after adjustment for all covariates (table 2, model C). Smaller, statistically insignificant effect sizes were estimated in the two models that did not control for time-varying potential confounding factors (eg, model B: −0.21 kg/m^2^, 95% CI −0.47 to 0.06). When the effects of switching to active travel and public transport were modelled separately, larger and statistically significant adjusted effect sizes were associated with switching to active travel between t0 and t2 (model D: −0.45 kg/m^2^, −0.78 to −0.11) and in the analysis restricted to participants who switched to active travel between t0 and t1 (model F: −0.59 kg/m^2^, −1.11 to −0.06). Effect sizes associated with switching from private motor transport to active travel also consistently became larger as participants with shorter baseline journeys were excluded from the analysis, rising to −0.75 kg/m^2^ among those switching to active travel with journey times >10 min to −2.25 kg/m^2^ for those >30 min (table 2, models G–I).

**Table 2:** Associations between change in mode of travel to work and change in body mass index

Analysis 1: Impact of switching from private motor transport to active travel or public transport									
Model characteristics^a^	Minimally adjusted models		Maximally adjusted models						
	All participants				As models C and D, except restricting the exposed group to participants who switched between t0 and t1		As model D, except restricting analysis to participants with longer commuting times at t0		
							>10 min	>20 min	>30 min
	Model A	Model B	Model C	Model D	Model E	Model F	Model G	Model H	Model I
Switch from private motor to public transport or active travel	−0.18	−0.21	−0.32*	N/a	−0.33	N/a	N/a	N/a	N/a
	(−0.45 to 0.0)	(−0.47 to 0.06)	(−0.60 to −0.05)		(−0.76 to 0.09)				
Switch from private motor to public transport				−0.12		0.12	−0.20	−0.23	−0.42
				(−0.55 to 0.30)		(−0.57 to 0.80)	(−0.67 to 0.27)	(−0.75 to 0.29)	(−1.05 to 0.22)
Switch from private motor to active travel				−0.45**		−0.59*	−0.75**	−1.64***	−2.25***
				(−0.78 to −0.11)		(−1.11 to −0.06)	(−1.23 to −0.28)	(−2.35 to −0.94)	(−3.33 to −1.18)
Observations	3269		3253		3144		2244	1289	752

Analysis 2: Impact of switching to private motor transport from active travel or public transport									
Model characteristics†	Minimally adjusted models		Maximally adjusted models						
	All participants				As models L and M, except restricting the exposed group to participants who switched between t0 and t1		As model M, except restricting analysis to participants with longer commuting times at t0		
							>10 min	>20 min	>30 min
	Model J	Model K	Model L	Model M	Model N	Model O	Model P	Model Q	Model R
Switch to private motor from public transport or active travel	0.34** (0.06 to 0.62)	0.33* (0.04 to 0.62)	0.34* (0.05 to 0.64)	N/a	0.37 (0.00 to 0.75)	N/a	N/a	N/a	N/a
Switch to private motor from public transport				0.46* (0.06 to 0.86)		0.44 (−0.10 to 0.98)	0.51* (0.06 to 0.96)	0.61* (0.13 to 1.1)	0.35 (−2.22 to 0.93)
Switch to private motor from active travel				0.26 (−0.09 to 0.62)		0.33 (−0.13 to 0.79)	0.39 (−0.14 to −0.93)	0.52 (−0.19 to 1.22)	0.52 (−0.53 to 1.58)
Observations	787		785		658		500	342	239

Values tabulated are β-coefficients and 95% CIs.

*p<0.05, **p<0.01 and ***p<0.001.

†See figure 2 for details of the variables and samples used in each statistical model. n/a, Not applicable.

#### Attrition bias and missing values bias

Significant differences in the characteristics of individuals, notably in terms of age, gender, income and baseline BMI were identified when comparing participants in the original BHPS sample with those retained in the analytical sample (see online supplementary appendix).

### Analysis 2: Switching from active travel or public transport to private motor transport

Of 787 individuals included in this analysis, 268 were in the exposed group. Of these, 156 switched from active travel (most often walking, n=121) and 112 from public transport (most often bus or coach, n=73). Again, switchers were significantly younger on average than non-switchers (eg, for active travel: 35.1 vs 41.2 years at t0, table 1), but other differences in baseline working hours, income, education, children, health status, mean BMI and obesity status were not significant. Car access was more prevalent among those who switched from active travel at t0 and t2 and also among those who switched from public transport at t2. Those who switched from active travel were significantly less likely than either non-switchers or those who switched from public transport to hold a professional or managerial occupation (eg, 24.4% for switchers from active travel vs 34.5% for non-switchers) and more likely to undertake weekly LTPA (74.4% vs 64.7%), and had a shorter mean commute time (13.7 vs 27.4 min at t0) which increased after switching to private motor transport (18.0 min at t2). In contrast, those who switched from public transport had a longer mean commute time (42.4 min at t0) which was reduced after switching to private motor transport (29.5 min at t2).

#### Effect on BMI

Switching from active travel or public transport to private motor transport was associated with a significant increase in BMI of 0.34 kg/m^2^ (0.05 to 0.64) after adjustment for all covariates (table 2, model L). When the effects of switching from active travel and public transport were modelled separately, a statistically significant adjusted effect size was associated with switching from public transport (model M: 0.46 kg/m^2^, 0.06–0.86). Statistically significant effects were not observed in the models restricted to participants who switched between t0 and t1.

## Discussion

### Principal findings

Our observation that switching from private motor transport to active travel or public transport was associated with a reduction in BMI, even in a relatively short-time period of under 2 years, suggests that a shift in the proportion of commuters using more active modes of travel could contribute to efforts to reduce population mean BMI. While previous studies have demonstrated cross-sectional associations between BMI and mode of travel to work, to the best of our knowledge this is the first study using cohort data from a longitudinal study of nationally representative households to link changes in BMI with changes in the usual main mode of travel to work. Combined with other potential health, economic and environmental benefits associated with walking, cycling and public transport,^5^ ^7^ ^8^ ^26–30^ these findings add to the case for interventions to promote the uptake of these more sustainable forms of transport.^2^ ^4^ ^31^ If large numbers of people could be enabled to take up active travel to work, for example through environmental and policy interventions in the transport and planning sectors, the benefits for population health may be larger than those of alternative interventions targeted at producing larger individual health benefits for relatively small numbers of people.^32^

#### Switching to active travel

We found significant negative associations between change in BMI and switching from private motor transport in models that accounted for the uptake of active travel and public transport both together (model C) and separately (model D). The case for causal inference is further strengthened by three key findings. First, we found a statistically significant effect in the analysis restricted to participants who switched to active travel between t0 and t1 (model F) in which the exposure is more likely to have temporally preceded the outcome. Second, we found stronger effect sizes when participants with shorter commutes were excluded from the analysis (models G–I), which is indicative of a dose–response relationship. Third, significant positive associations were observed in a separate sample of commuters who switched in the opposite direction (Models J–L).^33^ The direction and size of effects observed in this study are comparable to those of recent cross-sectional analyses of UK commuters which showed negative associations between BMI and walking (eg, −0.48 kg/m^2^, 95% CI −0.70 to −0.25)^5^ and cycling (−0.97 kg/m^2^, −1.30 to −0.63)^5^ compared with private motor transport,^5^ ^6^ and with those reported in reviews of interventions to promote walking,^34^ including a review of 23 randomised controlled trials which reported an average reduction in BMI of −0.53 kg/m^2^ (−0.72 to −0.35) associated with uptake of regular walking.^35^

The finding that participants who switched to active travel were, on average, from lower income households, less likely to be educated to degree-level or higher and more likely to work part-time than other participants in the study (see table 1) could be indicative of the potential for interventions in the transport and planning sectors to support strategies to reduce health inequalities.^5^ ^36^

#### Switching to public transport

The significant negative association observed between change in BMI and switching from private motor transport to active travel or public transport (model C), and the significant positive association with switching from public transport to private motor transport (models J–M), supports the implications of existing studies showing that public transport users can undertake meaningful levels of physical activity when accessing stations or stops.^5^ ^18^ ^19^ ^37–39^ The cross-sectional UK studies referred to above also identified an association between BMI and public transport use compared with private motor transport (eg, −0.24 kg/m^2^).^5^ ^6^ Nevertheless, we did not observe significant associations in our analyses of switching from private motor transport which accounted for public transport separately from active travel (models D and F). This may reflect important differences between bus and rail travel—for example, that rail passengers walk further on average to access stops than bus passengers^5^ ^18^ ^19^ ^37–39^—which could not be adequately explored in this study because of small sample sizes. Large differences were also identified in the socioeconomic characteristics of participants who switched to rail travel compared with those who switched to bus travel (eg, mean household income: £45 113 vs £25 959). While rail travel in Great Britain has grown at a much faster rate than road traffic or bus travel in recent years,^40^ future studies could explore the size and distribution of benefits associated with these changes and their implications for strategies to reduce health inequalities.

### Strengths and limitations

In contrast to existing cross-sectional studies, the main strength of this study lies in its use of cohort data from a longitudinal study of nationally representative households to examine associations between changes in mode of travel to work and changes in BMI over time. This study design was also able to account for a number of potential time-varying confounding variables (such as substantial changes in health and income). Nevertheless, because the BMI outcome variable was not reported at t1 we cannot be sure that the changes in mode of travel preceded the changes in BMI. A further limitation is that BMI was based on self-reported measures, which are typically biased when compared with direct measurements.^41^ However, our reliance on within-individual changes over a 2-year period was probably subjected to a lower risk of bias than might be the case for between-individual comparisons. Since the main exposure of interest was the usual main mode of travel to work, the analysis could not take full account of multimodal trips such as park-and-ride, or other trips undertaken during leisure or work time. Missing data, attrition (see online supplementary appendix and figure 1) and the differences in some observed characteristics between exposed and unexposed groups (see table 1) appears to have introduced some bias; some potential time-varying confounding variables, including other physical activity and dietary behaviours, were unobserved; and the relatively short follow-up time precluded the examination of longer term health effects. While small sample sizes and limited within-individual variation prevented the use of more advanced analytical approaches such as fixed effects models or instrumental variables, these could contribute to mitigating the impact of various sources of bias and might therefore be considered in future research.^25^

## Conclusion

This study has extended existing literature on the health benefits of active travel by providing longitudinal evidence from a national survey of a relationship between switching to and from more active modes of travel to work and modest changes in BMI.

**What is already known on this subject**

- Previous cross-sectional studies have shown that commuters who used active travel or public transport had significantly lower body mass index (BMI) than their counterparts who used private motor transport. However, no longitudinal study has used cohort data from a nationally representative survey to explore the impact on individual-level BMI of switching between different modes of travel.

**What this study adds**

- This study used cohort data from the British Household Panel Survey and identified a statistically significant net reduction in body mass index over a 2-year period among commuters who switched from private motor transport to active travel or public transport. The results provide more robust support for causal inference than existing cross-sectional studies and strengthen the case for policymakers to promote population health by incentivising walking or cycling.

## Supplementary Material

Web appendix
