# Supplementary material for: Impact of changes in mode of travel to work on changes in body mass index: evidence from the British Household Panel Survey
Source: J Epidemiol Community Health. 2015 May 7;69(8):753–61. doi: 10.1136/jech-2014-205211 (PMC4515986; doi:10.1136/jech-2014-205211)
Supplement: Web appendix [file jech-2014-205211-s1.pdf]

Appendix: An assessment of missing values and attrition bias

The table shows descriptive statistics and group comparisons for samples of participants before and after a sample restriction was imposed. The test for missing values compared participants who remained in the sample (n=7,471) with those excluded because they did not report BMI at t0 (n=339). The test for attrition bias compared individuals who remained in the sample (n=6,634) with those excluded from the analysis because they were no longer in the dataset at t2 (n=837). The results show significant differences in the characteristics of individuals (e.g. in terms of age, gender, income and BMI) between groups, indicating that attrition and missing values are likely to be a source of bias in the results.

Characteristic (at t0)	Test for missing values (height and weight data) bias (See [a] in Figure 1)			Test for attrition bias (See [b] in Figure 1)		
	% or mean		p[b]	% or mean		p[b]
	Participants retained in the sample	Participants excluded from analysis due to missing BMI at t0		Participants retained in the sample	Participants excluded from analysis due to leaving the dataset before t2	
n[a]	7,471	339		6,634	837	
Age (mean years)	39.6	37.2**	**0.001**	35.6	40.1	**<0.001**
Male[c]	50.4	19.8***	**<0.001**	52.1	50.2	0.298
Professional or managerial occupation[c]	39.2	33.9	0.052	37.5	39.4	0.296
Full time work[c]	78.0	66.7***	**<0.001**	79.9	77.8	0.152
Works at night time[c]	2.1	2.9	0.319	2.3	2.1	0.785
Household income (mean £s)	31,126.8	29,021.5*	**0.011**	29,417.9	31,342.4	0.008
Education: Degree or higher qualification[c]	19.2	13.6**	**0.009**	20.0	19.1	0.576
One or more children in the household[c]	19.6	30.7***	**<0.001**	14.1	20.3	**<0.001**
Lives in London or South-East England[c]	13.9	13.3	0.737	16.1	13.6	0.050
BMI (mean kg/m^2)	n/a	n/a	n/a	25.2	26.0	**>0.001**
WHO-classified overweight[c]	n/a	n/a	n/a	47.2	52.9	**0.002**
‘Poor’ or ‘very poor’ self-assessed health[c]	4.8	6.8	0.091	5.0	4.7	0.716
Self-reported smoker[c]	25.9	23.3	0.278	31.7	25.2	**<0.001**
More than 3 annual hospital visits [a c]	11.4	15.1*	**0.039**	10.3	11.6	0.268
More than 6 annual primary care visits [a c]	10.7	13.3	0.130	10.0	10.8	0.510
One or more cars in household[c]	91.2	86.1**	**0.001**	88.1	91.6	**0.001**
Number of cars in household (mean)	1.6	1.5	0.122	1.5	1.6	0.005
Commute time (mean minutes)[a]	23.8	20.4**	**0.002**	24.8	23.7	**0.031**
At least weekly LTPA [a c]	59.0	52.8*	**0.023**	58.5	59.1	0.735
At least weekly gardening [a c]	22.4	16.8*	**0.015**	15.1	23.3	**<0.001**
At least weekly eating out [a c]	18.3	15.3	0.172	21.3	17.9	**0.016**

* p<0.05, ** p<0.01, *** p<0.001

[a] Sample sizes used in a small number of cases was less than shown since values were not reported for some variables for all individuals.

[b] The results of Chi-squared tests (or Mann-Whitney tests for number of cars, age, income and commute time, or student's t-tests for BMI), where the null hypothesis was that the difference between the two groups was equal to zero.

[c] Binary variables were created as described in Table 2.
